# Characterizing Listener Engagement with Popular Songs Using Large-Scale Music Discovery Data

**DOI:** 10.3389/fpsyg.2017.00416

**Published:** 2017-03-23

**Authors:** Blair Kaneshiro, Feng Ruan, Casey W. Baker, Jonathan Berger

**Affiliations:** ^1^Center for Computer Research in Music and Acoustics, Stanford UniversityStanford, CA, USA; ^2^Shazam Entertainment, Ltd.Redwood City, CA, USA; ^3^Department of Statistics, Stanford UniversityStanford, CA, USA

**Keywords:** Shazam, popular music, music discovery, multimedia search, music information retrieval, musical engagement, social media

## Abstract

Music discovery in everyday situations has been facilitated in recent years by audio content recognition services such as Shazam. The widespread use of such services has produced a wealth of user data, specifying where and when a global audience takes action to learn more about music playing around them. Here, we analyze a large collection of Shazam queries of popular songs to study the relationship between the timing of queries and corresponding musical content. Our results reveal that the distribution of queries varies over the course of a song, and that salient musical events drive an increase in queries during a song. Furthermore, we find that the distribution of queries at the time of a song's release differs from the distribution following a song's peak and subsequent decline in popularity, possibly reflecting an evolution of user intent over the “life cycle” of a song. Finally, we derive insights into the data size needed to achieve consistent query distributions for individual songs. The combined findings of this study suggest that music discovery behavior, and other facets of the human experience of music, can be studied quantitatively using large-scale industrial data.

## 1. Introduction

Discovering new music is a popular pastime, and opportunities for music discovery present themselves throughout everyday life. However, relatively little is known about this behavior and what drives it. In a recent interview study, Laplante and Downie (2011) found that the active, deliberate search for music information—whether finding new music or information about music—is generally considered both useful and intrinsically enjoyable. In an earlier diary study, however, Cunningham et al. (2007) report that the majority of exposures to new music occur in passive encounters—that is, when a listener was not actively seeking to discover new music. Furthermore, while participants in that study reacted positively to over 60% of their encounters with new music, they also reported that passive music encounters were difficult to act upon in the moment. Since the publication of that study, the rise of mobile services and ubiquitous internet now facilitate instantaneous music discovery during everyday life, whether music is actively sought or passively encountered. Accompanying the widespread use of such services is an unprecedented volume of user data bearing potential insights into where and when people discover music, as well as what music they choose to discover. These data surpass what can be collected through controlled laboratory or ethnographic studies in terms of size, scope, and ecological validity.

In recent years, industrial user data reflecting a variety of musical behaviors—including but not limited to social sharing, consumption, and information seeking—have been utilized in music informatics research. Twitter, being freely available for aggregation, currently serves as the most common source of data and has been used to explore a variety of topics including artist and music similarity (Schedl, 2010; Schedl et al., 2014), music recommendation (Zangerle et al., 2012; Pichl et al., 2014, 2015), geographical attributes of music consumption (Schedl, 2013; Moore et al., 2014), and hit prediction (Kim et al., 2014; Zangerle et al., 2016). Music consumption and sharing has also been approached using Spotify URLs shared via Twitter (Pichl et al., 2014, 2015) and music download data from the MixRadio database (Bansal and Woolhouse, 2015). A number of these studies have contributed or made use of publicly available research corpuses, including the Million Musical Tweets Dataset, containing temporal and geographical information linked to music-related tweets (Hauger et al., 2013); the continually updated #nowplaying dataset of music-related tweets (Zangerle et al., 2014); and Gracenote's GNMID14 dataset, which includes annotated music identification matches (Summers et al., 2016).

In the present study, we explore large-scale music discovery behavior using query data from the audio identification service Shazam^1^. In particular, we investigate whether the timing of audio identification queries within a song can be related back to specific musical events. We aggregate and analyze a large collection of Shazam query *offsets*—that moment in a song when a user initiates a query—over a set of massively popular songs. We first verify that the distribution of query offsets is not uniform but in fact varies over the course of a song. Next, we show that the overall shape of a query offset histogram also varies over the “life cycle” of a hit song, with more queries occurring toward the start of a song once the song has achieved widespread popularity. We then demonstrate that salient musical events—such as the start of a song, onset of vocals, and start of first chorus—are followed by a rise in query activity. We conclude with an assessment of histogram consistency as a function of data size in order to determine what constitutes a sufficient data size for this type of analysis. The findings from this study provide first insights into the types of musical events that engage listeners at a large scale, compelling them to take action to obtain more information about a piece of music. To our knowledge, this is the first time that engagement with specific musical events has been studied with an ecologically valid, large-scale dataset. Findings from this study will advance knowledge of consumption of popular music, information seeking about music, and—more broadly—how and when a large audience chooses to engage with music in their environment. Finally, to promote further research on music discovery, the dataset of over 188 million Shazam queries analyzed in this study is made publicly available.

## 2. Materials and methods

### 2.1. Audio content recognition with Shazam

Shazam is a service that returns the identity of a prerecorded audio excerpt—usually a song—in response to a user query. Over 20 million Shazam queries are performed each day by more than 100 million monthly users worldwide; incoming queries are matched over a deduplicated catalog comprising over 30 million audio tracks. Shazam's audio recognition algorithm is based on fast combinatorial hashing of spectrogram peaks, and was developed with real-world use cases in mind. As a result, Shazam's performance is robust to noise and distortion; provides fast performance over a large database of music; and offers a high recognition (true-positive) rate with a low false-positive rate (Wang, 2003).

**Figure 1:**
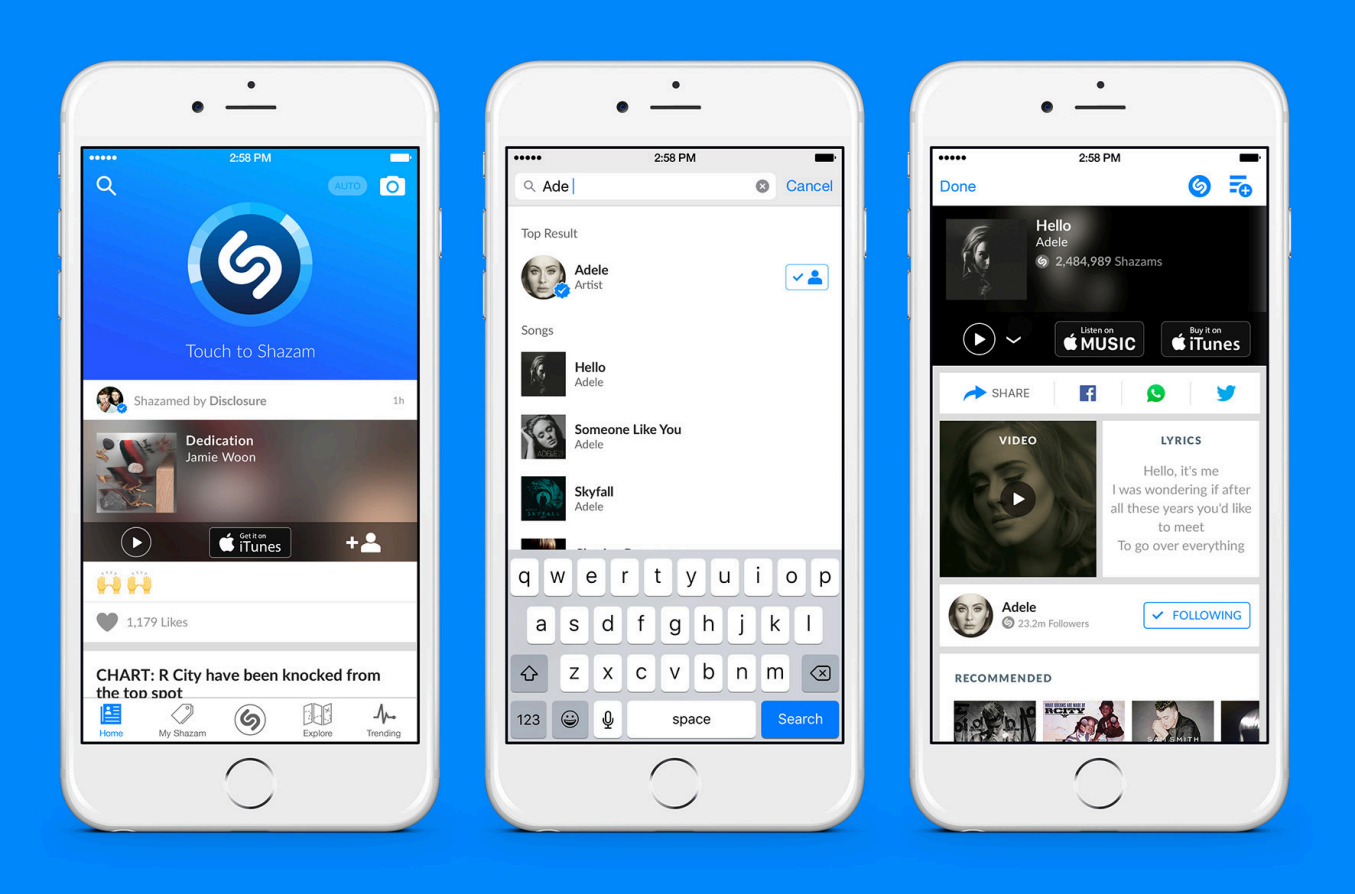
**Shazam application screenshots**. Shazam audio queries are typically initiated from a mobile device. **(Left)** Upon loading the application, the Shazam icon is prominently displayed on the main screen. **(Center)** Queries can also be initiated through a text search. **(Right)** A successful audio query or selection from text query results returns the track page for the song of interest. Information returned to the user on the track page includes basic metadata about the song, as well as related media including the music video and lyrics when available. The Shazam logo is ubiquitously displayed as users navigate the application; thus, new queries can be initiated at any time. Image used with permission.

Shazam queries typically involve a single button press once the application is loaded. For queries initiated from mobile devices,^2^ the user loads the Shazam application and pushes a prominently displayed Shazam icon on the main screen (Figure 1, left). The ambient acoustical signal is recorded through the device microphone, converted to an audio fingerprint, and matched. If the query is matched successfully, the match result is then displayed on the device screen. In the most common use case of song identification, the application will return a variety of metadata (Figure 1, right) including song title and artist; total number of Shazam queries for the track identifier (“trackid”) corresponding to the match; and options for sharing the query result (e.g., through social media or text message). Oftentimes the query result will also include links to third-party services to purchase or stream the song; links to watch the song's music video on YouTube; an option to view song lyrics; and music recommendations. The Shazam icon is displayed somewhere onscreen at all times; thus, users can easily initiate new queries without having to return to the home screen of the application. Selected platforms also offer an “Auto Shazam” feature, which prompts the application to listen and attempt audio matches continuously in the background. Users can additionally retrieve track results through text searches (Figure 1, center).

The audio matches, metadata, and other features listed above represent data returned to users. Each query additionally generates a collection of data stored internally to Shazam, including date and time of the query; location information if the user has agreed to share it; the returned track and other candidate tracks that were not returned; metadata associated with the returned track; device platform (e.g., iOS, Android); language used on the device; installation id of the application; and the length of time the query took to perform. Importantly, Shazam also stores the query “offset,” which is the time stamp of the initiation of the query relative to the start of the returned track. In other words, the offset tells us when in a song the user performed the query. The present analysis utilizes query offsets and dates.

### 2.2. Dataset

#### 2.2.1. Song set

As this study is a first quantitative analysis of Shazam query offsets, we chose to limit the number of songs used for analysis, but to select songs that would each offer an abundance of Shazam queries while also reflecting a widespread listening audience. For these reasons, we chose as our song set the top 20 songs from the Billboard Year End Hot 100 chart for 2015, which lists the most popular songs across genres for the entire year, as determined by radio impressions, sales, and streaming activity^3^. An additional advantage of selecting songs from this particular chart is that the Billboard Hot 100 chart is released weekly; therefore, our analyses can probe music discovery behavior at specific stages of song popularity. Billboard charts in general are considered a standard industry measure of song popularity, and weekly Billboard Hot 100 charts in particular have been used as a benchmark of song popularity in a number of previous studies (Kim et al., 2014; Nunes and Ordanini, 2014; Nunes et al., 2015; Zangerle et al., 2016).

The set of songs is summarized in Table 1. The 15th-ranked song on the Billboard chart (“Bad Blood” by Taylor Swift Feat. Kendrick Lamar) was excluded from analysis due to a known problem with the query data. We therefore include the 21st-ranked song in the set in order to have a set totaling 20 songs.

**Table 1:** **Song and dataset information**.

**Rank**	**Title**	**Artist**	**Length (s)**	**Shazam query offsets**	
				**% usable**	**# usable**
1	Uptown Funk!	Mark Ronson Feat. Bruno Mars	270	98.57	13,855,245
2	Thinking Out Loud	Ed Sheeran	282	98.97	17,142,656
3	See You Again	Wiz Khalifa Feat. Charlie Puth	230	98.73	12,522,399
4	Trap Queen	Fetty Wap	223	98.77	6,072,939
5	Sugar	Maroon 5	236	98.92	5,811,731
6	Shut Up and Dance	Walk the Moon	200	98.47	5,034,637
7	Blank Space	Taylor Swift	232	98.11	6,764,128
8	Watch Me	Silento	186	96.99	4,463,863
9	Earned It (Fifty Shades of Grey)	The Weeknd	252	98.66	7,514,440
10	The Hills	The Weeknd	243	99.08	8,657,473
11	Cheerleader (Felix Jaehn Remix)	OMI	182	96.84	17,933,224
12	Can't Feel My Face	The Weeknd	214	99.34	8,675,375
13	Love Me Like You Do	Ellie Goulding	251	99.56	9,925,090
14	Take Me to Church	Hozier	242	98.82	15,854,482
16	Lean On	Major Lazer & DJ Snake Feat. M0	177	99.10	19,974,795
17	Want to Want Me	Jason Derulo	208	98.89	9,885,505
18	Shake It Off	Taylor Swift	220	95.90	3,162,707
19	Where Are Ü Now	Skrillex & Diplo with Justin Bieber	251	99.44	7,639,899
20	Fight Song	Rachel Platten	205	99.23	4,359,870
21	679	Fetty Wap Feat. Remy Boyz	197	98.71	3,020,785
				TOTAL	188,271,243

*Shazam queries corresponding to 20 top-ranked songs from the Billboard Year End Hot 100 chart for 2015 were analyzed in the study. Song lengths are rounded up to the nearest second. The percent usable and number of usable queries reflect the cleaned datasets. Song 15 is omitted from analysis*.

##### 2.2.1.1. Song metadata

As the selected set of songs all achieved widespread popularity, it was possible to aggregate additional information about the songs from a variety of public sources. We obtained release dates from each song's Wikipedia page. Peak Billboard chart dates were obtained from the Billboard Hot 100 weekly charts and verified against Wikipedia when possible. For songs that held their peak chart position for multiple weeks, we used the date of the first week that the peak position was reached.

To identify the most “correct” version of the audio for each song, we followed the Amazon purchase link, when it was available, from the Shazam track page corresponding to the primary trackid of the song. If the Amazon link was missing or led to a clearly incorrect destination, we located the song on Amazon manually or through an alternate Shazam trackid. We purchased digital versions of all tracks from their resolved Amazon destinations, and then verified the song lengths against primary Spotify results when possible.

##### 2.2.1.2. Coding of salient musical events

Portions of our analysis focus on the onset of vocals and onset of the first occurrence of the chorus. While the songs analyzed here broadly represent “popular music,” assigning conventional pop-song labels, such as verses and choruses, to the structural elements of the songs proved somewhat challenging and subjective. Therefore, for an objective identification of chorus elements within each song, we used lyrics from the Genius website,^4^ which are both fully licensed^5^ and annotated with structural song-part labels such as “Verse” and “Chorus.” For the first onset of vocals, we used the audio timing linked to the first occurrence of labeled (e.g., “Verse” or “Bridge”) content in the lyrics, ignoring “Intro” content. For the first occurrence of the chorus, we identified the timing of the audio corresponding to the first instance of “Chorus” or “Hook” material in the lyrics. These times are not necessarily disjoint for a given song—e.g., the first entrance of vocals could be an instance of the chorus.

Additional metadata for the song set, including Shazam and Amazon track identifiers, release and peak Billboard dates, and onset times of vocals and choruses, are included in the Table S1.

#### 2.2.2. Shazam data aggregation and preprocessing

For the selected songs, we aggregated worldwide Shazam query dates and offsets from the Shazam database over the date range January 1, 2014 through May 31, 2016, inclusive. All but one song were released after January 1, 2014, and songs peaked on Billboard between September 6, 2014 and October 31, 2015. Therefore, we consider this date range representative of a song's journey through the Billboard charts. Aggregated data include audio queries only—no text queries—and do not include Auto Shazam queries or queries performed through the desktop application.

Offset values are given in seconds with sub-millisecond precision. Dates are resolved by day, based on GMT timestamps. To clean the data, we removed incomplete queries (missing date or offset values) as well as queries with offsets less than or equal to zero, or greater than the length of the corresponding audio recording. We did not exclude queries whose date preceded the release date, as listed release dates for songs as singles could come after the release date for an album on which the song was included.

The number of usable queries per song ranged from 3,020,785 to 19,974,795, with a median value of 8,148,686 queries. Between 95.90 and 99.56% of the original number of queries for each song were usable after data cleaning. In total, the dataset comprises 188,271,243 queries across the 20 songs. The cleaned datasets are publicly available for download in .csv format from the Stanford Digital Repository (Shazam Entertainment, Ltd., 2016)^6^.

### 2.3. Analysis

All data preprocessing and analyses were performed using R software, version 3.2.2 (R Core Team, 2015).

#### 2.3.1. Tests of uniformity

As the present study rests on the assumption that volumes of Shazam queries are higher at some points of a song than others, our first analysis was to determine whether the volume of query offsets for a given song indeed varies over time. To address this first question, we performed two-sided Kolmogorov-Smirnov tests (Conover, 1971) on the distributions of offsets for each song, comparing each distribution of offsets to a uniform distribution over the interval [0, *songLength*]. Under the null hypothesis of uniformly distributed query offsets, Shazam queries would be equally likely to occur at any point during a song, precluding further exploration of musical events that drive peaks in the query offset histograms. Due to the possibility of ties with our present data size, we added a small perturbation to each offset (uniformly distributed random variables over the interval [−0.000005, 0.000005]) before performing the tests.

#### 2.3.2. Assessing changes in histogram shape

Our second question concerned changes in histogram shape over time. Anecdotal analyses of Shazam query offsets have suggested that once a song becomes popular, the distribution of query offsets shifts closer to the beginning of the song.

To approach this problem quantitatively required both a temporal metric of song popularity and a definition for what portion of a song constitutes its “beginning.” To address the first point, we selected three points of interest in the life cycle of each song: The song's release date; the date of its peak on the Billboard Hot 100 chart; and the end dates of the dataset. Ranges of time between these three events varied by song. Songs peaked on Billboard between 19 and 463 days after release, with a median release-to-peak delay of 127 days. The time range between peaking on Billboard and the last date in the dataset ranged from 213 to 633 days, with a median value of 374 days. Dates and latencies between dates are reported in Table S1.

For the second point, instead of choosing an arbitrary, fixed duration (e.g., 30 s) to denote the beginning of each song, we devised an analysis that would compare distributions over all possible beginning durations *d*_*b*_ using the following procedure. For each song, we first extracted the first 100,000 queries following release and peak Billboard dates, and the final 100,000 queries, by date, in the dataset. Following that, for *d*_*b*_ increasing from 1 to the length of the song in seconds, we performed Chi-squared tests of proportions on Billboard peak date vs. release date, end of dataset vs. release date, and end of dataset vs. Billboard peak date. Because we were specifically interested in assessing whether queries migrated toward the beginning of the song for the later set of queries, we performed one-sided tests with the alternative hypothesis being that the proportion of queries less than *d*_*b*_ was greater for the set of queries corresponding to the later time point.

Due to data size, the *p*-values resulting from these tests were generally so small as to be uninformative. Therefore, we focus on percentile Chi-squared statistics over increasing *d*_*b*_ for each song, and report these results across songs. This analysis comprises a total of 13,503 multiple comparisons (three comparisons per time point per song times 4,501 total time points across all songs). Therefore, as we do not correct here for multiple comparisons, we use a conservative significance threshold of *p* < 10^−10^, keeping us well under the statistical significance threshold of α = 0.01, had a Bonferroni correction been performed (Bonferroni, 1936; McDonald, 2014).

#### 2.3.3. Computing histogram slopes at salient musical events

For our third analysis, we wished to test the hypothesis that salient musical events drive a subsequent increase in query volume. For the present analysis we chose three salient structural events that were present in every song: Beginning of song, initial onset of vocals, and initial onset of chorus/hook section.

We devised an exploratory analysis of the query offset volume around these musical events by focusing on offset histogram slopes following these events. As our previous analysis revealed a leftward shift in offset distributions for later dates, we used only the first 1,000,000 queries for each song (by date) for this computation. We first used local polynomial regression (Fan and Gijbels, 1996) to estimate histogram slopes over time for each song, with a temporal resolution of 1 s. We then converted each song's estimated histogram slopes to slope percentiles in order to bring the data to a more common scale across songs. As the timing of onset of vocals and chorus can vary from song to song, we extracted 15-s analysis windows starting from the onset of each event, and then for each event type (beginning, vocals, chorus) we aggregated the windows across songs so that the 15-s intervals were now aligned according to the onsets of the musical event of interest—similar to the approach taken by Tsai et al. (2014) in analyzing physiological responses at chorus onsets across a set of popular songs.

For each of the musical events of interest, we report the median of histogram slope percentiles over time across the songs, along with first and third quartiles. For reference, we also report results from the same analysis, using randomly selected window start times for each song.

#### 2.3.4. Data size and histogram consistency

Our final analysis examined the relationship between data size and histogram consistency. One reason for selecting massively popular songs was to have millions of queries to work with for each. But do the underlying distributions of the data require such large collections of queries, or is a smaller sample size sufficient?

To investigate this matter further, we assessed consistency of query offset distributions, computing histogram distance between disjoint data subsets of varying sample size for individual songs. For songs whose data comprised more than 8 million queries, we drew a random subsample of 8 million queries for the following analysis. On a per-song basis we randomly partitioned the collection of queries into two halves. For an increasing number of trials *n*_*i*_ from 1 to *nTotalTrials*/2, we normalized the cumulative histograms of the two halves into discrete probability densities (each summing to 1), and then used the total variation distance (Levin et al., 2009) to measure the distance between these two probability distributions. This partitioning procedure was repeated over 100 randomization iterations for each song. We then computed the mean output across randomization iterations for each song. We report the median, across songs, of these results.

## 3. Results

### 3.1. Distributions of query offsets are not uniform

**Figure 2:**
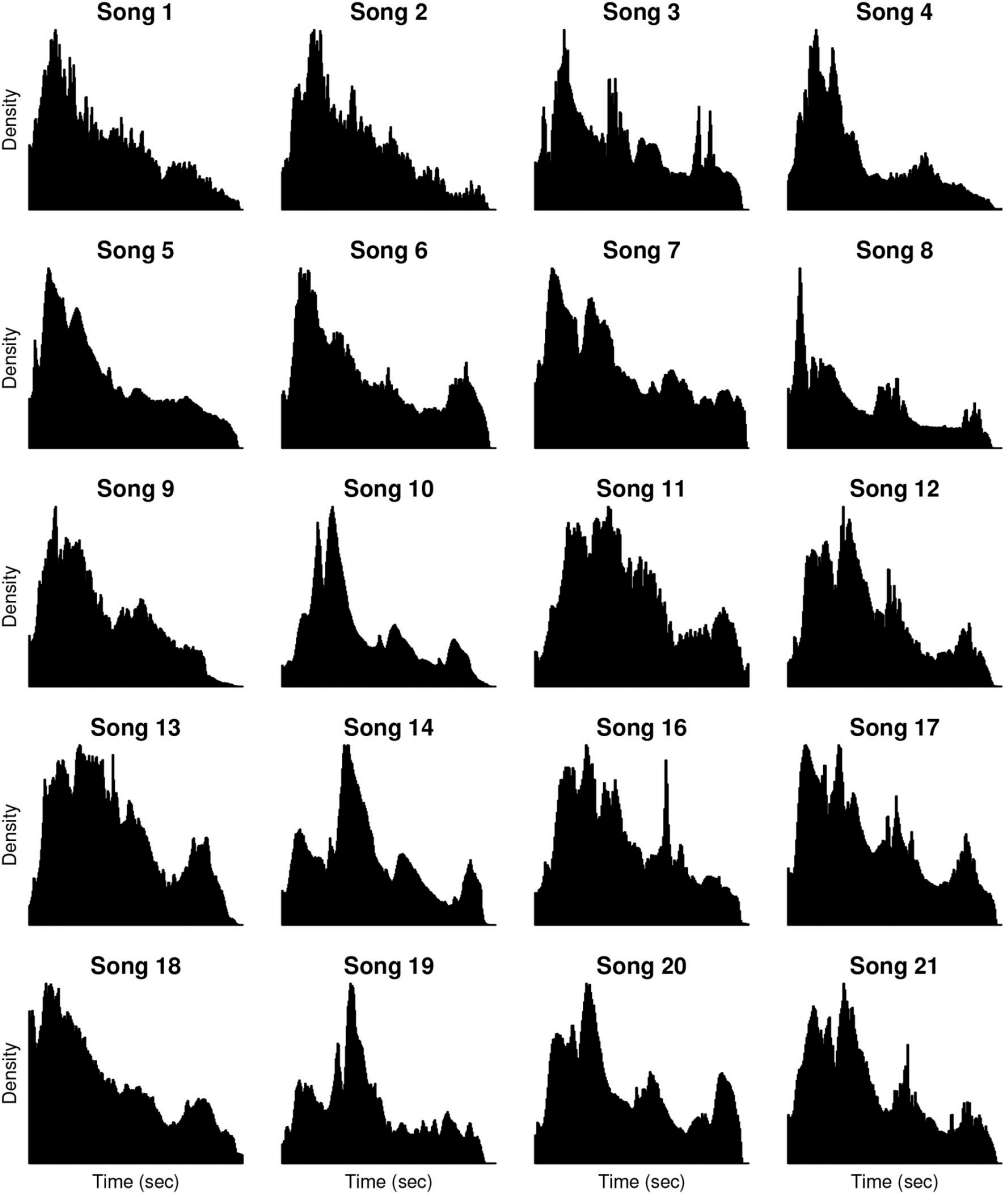
**Shazam query offset histograms**. Histograms are computed from the query offsets of the 20 hit songs analyzed in the study (summarized in Table 1). Each histogram presents the density of Shazam queries (y-axis) over time (x-axis) for a given song. Histograms are scaled to maximum density and song duration on a per-song basis. The number of queries per song ranges from 3,020,785 (Song 21) to 19,974,795 (Song 16), with a median of 8,148,686 queries per song.

For our first analysis, we assessed whether query offsets for a given song are uniformly distributed over time (implying no relationship between musical events and number of queries), or whether the volume of queries varies over the course of a song. Scale-free plots of the offset histograms are shown in Figure 2. By visual inspection, the histograms do not reflect uniform distributions of query offsets. Additionally, the timing, height, and shape of the histogram peaks vary from song to song. Results of the Kolmogorov-Smirnov tests of uniformity provide a quantitative validation of our observations, rejecting the null hypothesis with *p* < 10^−15^ for all songs (no correction for multiple comparisons).

### 3.2. Shapes of offset histograms change over time

**Figure 3:**
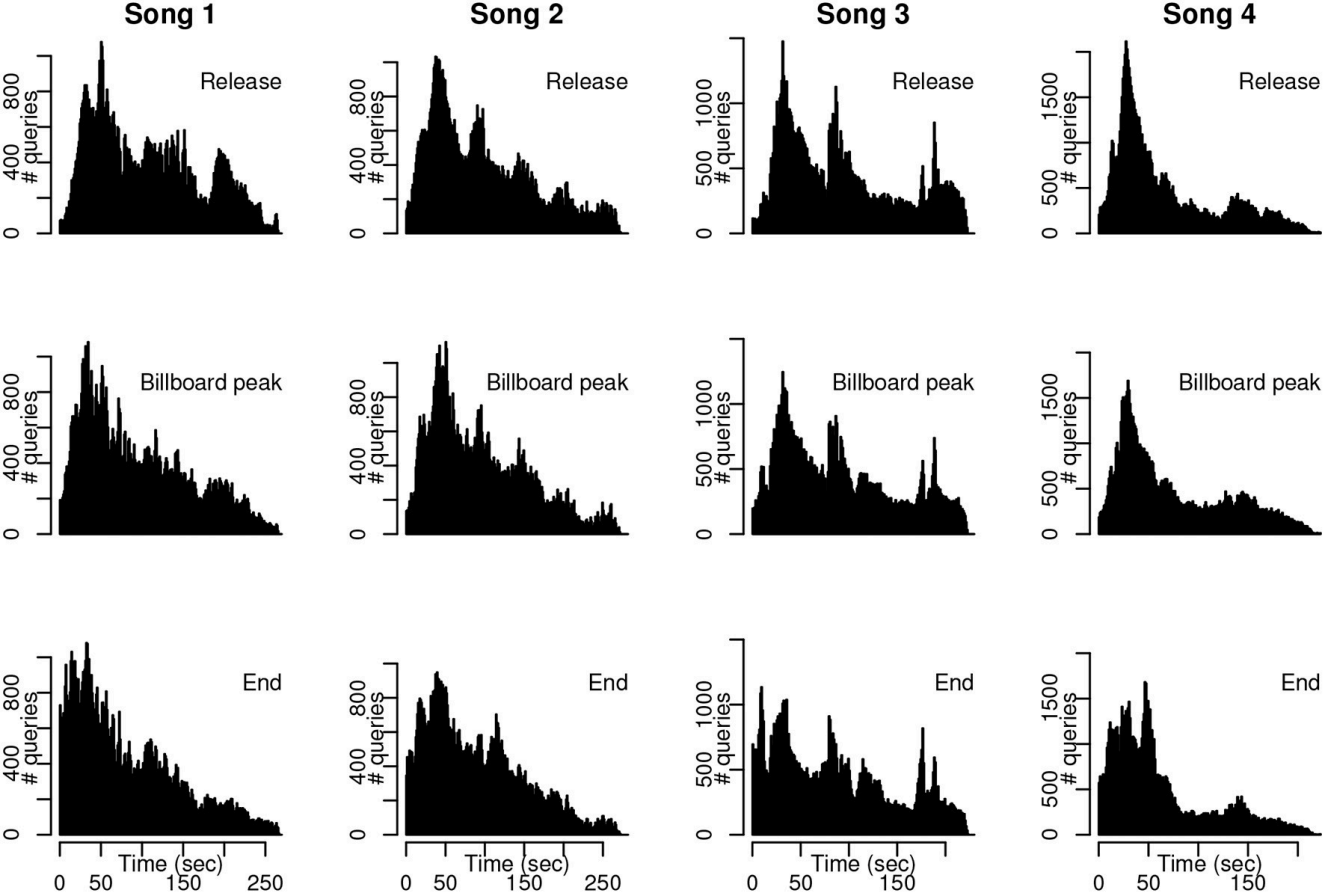
**Example histograms throughout hit song life cycle**. Subsampled query distributions at the time of song release, date of peak position on Billboard, and end of the dataset for four songs. The distribution of query offsets for the end dates in particular exhibit a pronounced shift toward the beginning of each song.

Our second question was whether the distribution of query offsets shifts toward the beginning of a song as the song moves through its hit life cycle—that is, whether users tend to perform the Shazam query earlier in a song once the song has attained, or dropped from, popularity. Query offset histograms around release date, peak Billboard date, and end of the dataset are shown for the first four songs in our song set in Figure 3 (plots for remaining songs are included in Figures S1–S4). Each subplot comprises 100,000 queries. The shift in the histogram toward the beginning of the song (left side of each plot) is evident for each of these songs, especially for the “End” subset of the dataset.

**Figure 4:**
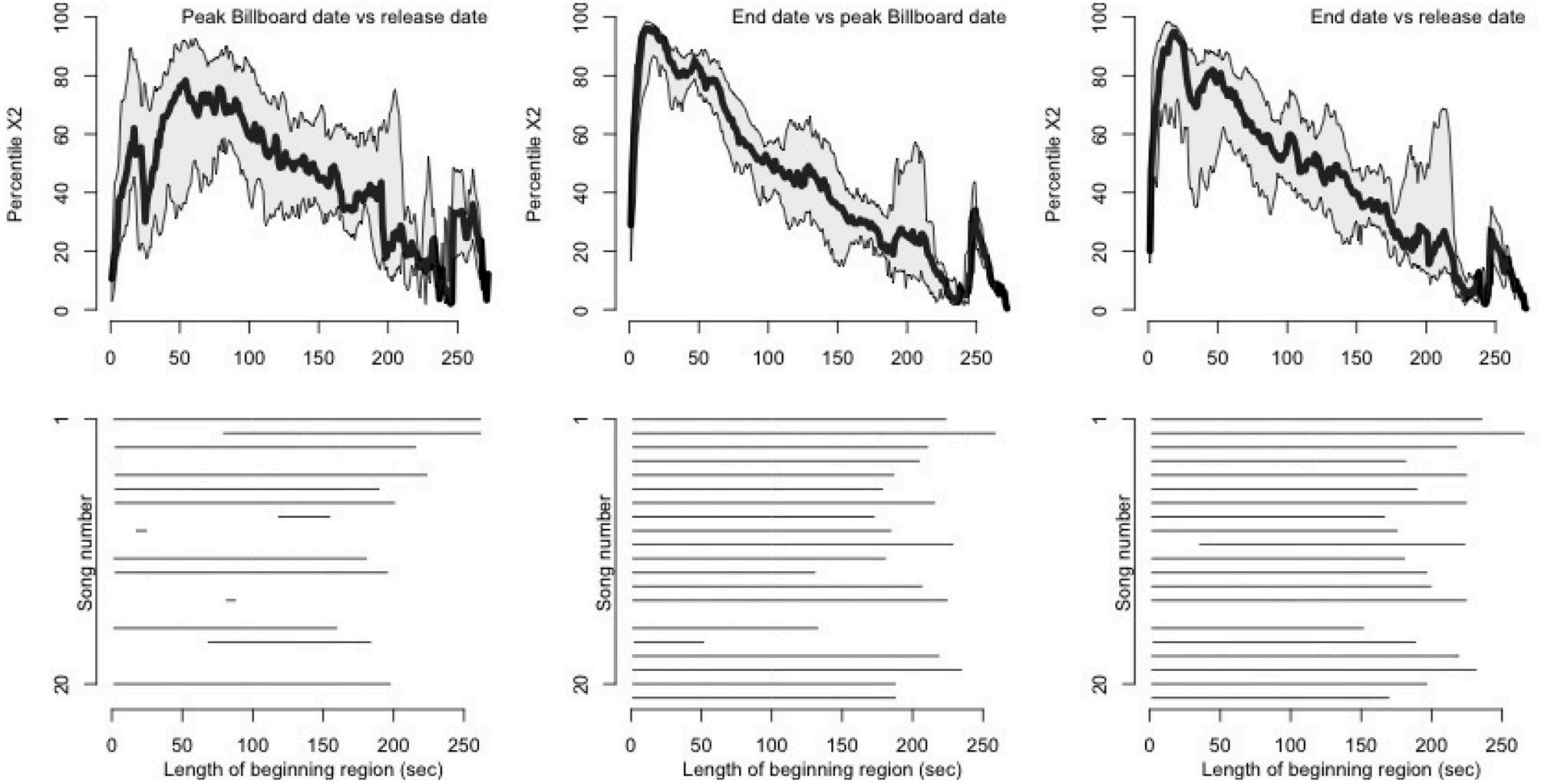
**Changes in histogram shape during the hit song life cycle**. We performed Chi-squared tests of proportions to assess whether distributions of query offsets migrate toward the beginning of the song as a song achieves popularity. One-sided tests compared each later-vs.-earlier pair of subsamples using a beginning window of increasing duration. **(Top)** Median percentile Chi-squared statistics, across songs, with first and third quartiles, for each pairwise test over beginning windows of increasing length. We converted statistics to percentiles on a per-song basis to impose a common scale across songs. For peak Billboard vs. end date and release date vs. end date, window lengths of around 50 s or less produce notably high Chi-squared statistics, demonstrating that query offsets for the latest dates are more concentrated at the beginnings of songs. **(Bottom)** Raster plot of beginning window lengths producing *p* < 10^−10^ in the tests of proportions for individual songs (no correction for multiple comparisons).

As a more quantitative assessment, we performed Chi-squared tests of proportions on sets of queries drawn from the time of song release, peak Billboard date, and final dates of the dataset. Chi-squared tests of proportions were performed over a beginning window of increasing duration to assess the size of the statistic when comparing pairs of life-cycle samples. Results are shown in Figure 4. In the top row of plots, percentile Chi-squared statistics (y-axis) as a function of beginning window length in seconds (x-axis) are plotted, with the median across songs shown in black, and first and third quartile of individual songs shown in gray. Median Chi-squared statistic percentiles are notably high at the beginnings of songs for end date vs. peak Billboard date (peaking at 13 s), and end date vs. release date (peaking at 19 s). This indicates that across songs, tests of proportions as to whether the later set of queries was distributed closer to the start of a given song returned consistently high Chi-squared statistics for the beginning portions of the songs.

More detail on individual songs is given in the bottom plots of Figure 4, which specifies the beginning window lengths that produced statistically significant Chi-squared statistics. Here, we see that nine of the songs in the set exhibited a constant migration of queries toward the start of the song from release date to peak Billboard date, and all 20 songs exhibited this shift when comparing queries from the peak Billboard date to those from the final dates in the dataset (recall that Song 15 was omitted from analysis). Comparing release date to end date, all but one song (Song 10) exhibit a leftward histogram shift when the first 30 s of the histogram are analyzed. Taken together, these results suggest that users do tend to perform queries earlier in a song for dates toward the end of the dataset, compared to dates around the song's release or peak on the Billboard Hot 100 chart.

### 3.3. Salient musical events drive increase in queries

**Figure 5:**
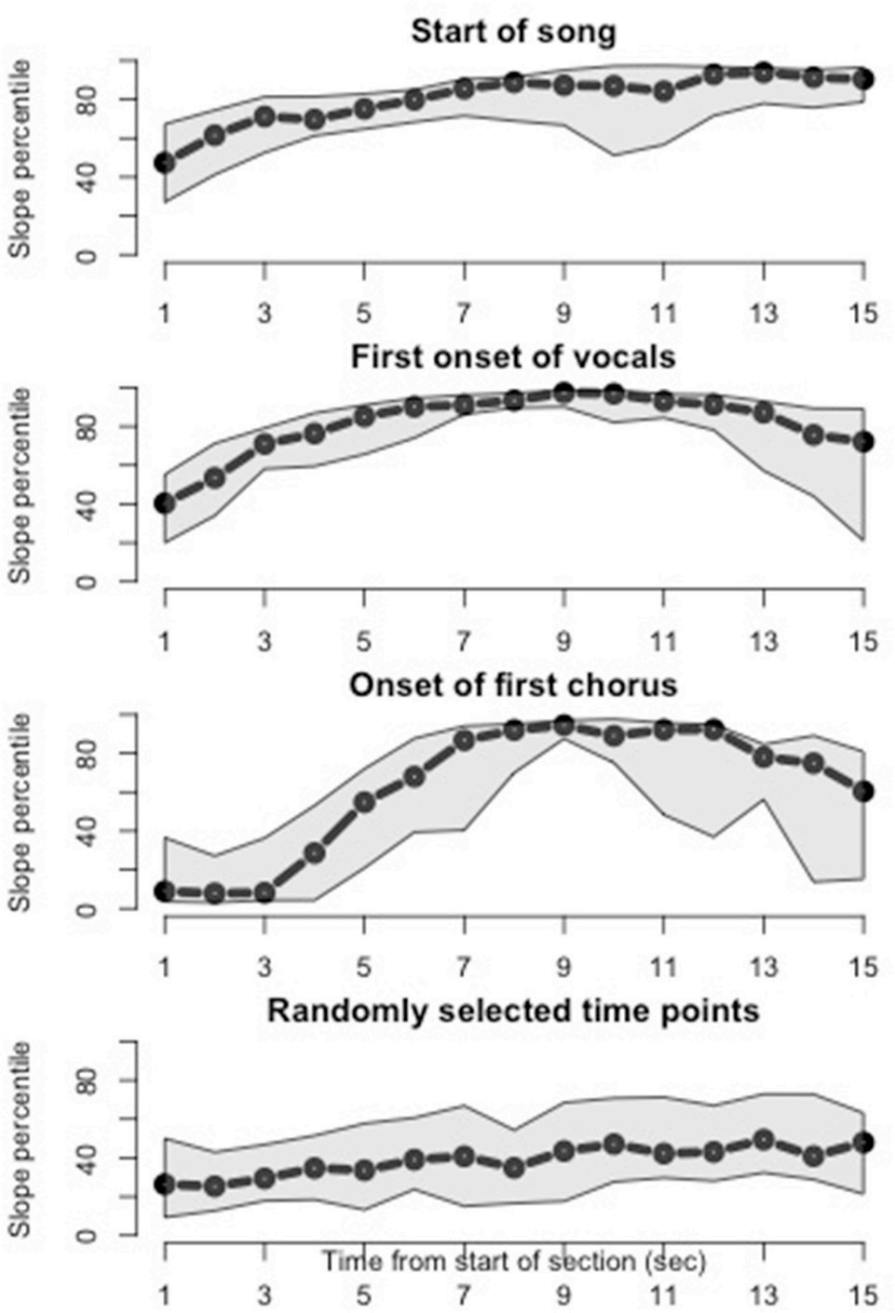
**Slopes of histograms after salient musical events**. Median (black) with first and third quartiles (gray) of histogram slopes across songs are plotted, time-locked to specific musical events. Histogram slopes for each song were converted to percentiles prior to plotting. Histogram slopes increase following the start of the song **(top plot)**, the first onset of vocals in the song **(second plot)**, and the onset of the first chorus **(third plot)**. In particular, histogram slopes are consistently high across songs around 9 s after the first onset of vocals and first onset of the chorus. **(Bottom plot)** When randomly selected time points, rather than salient musical events, are considered, the median histogram slope across songs over time peaks around the 50th percentile.

Our third analysis examined whether three salient musical events—the start of a song, the first onset of vocals, and the onset of the first chorus—would drive an increase in queries. This is a first step toward relating the histogram peaks, evident in Figure 2, to structurally salient musical events, and toward generalizing music discovery behavior across the songs, which vary in their timing and arrangement of shared musical events. The results of the histogram slope analysis by song part, summarized across songs, is shown in Figure 5. Each plot represents a 15-s window time-locked to the beginning, first onset of vocals, onset of first chorus, and random time point, respectively, across songs. Therefore, the x-axis of each plot is time, and the y-axis is percentile of histogram slope. The three structurally salient time points are all followed by notably high histogram slopes, representing an increase in query volume over time. As shown by the median measure across songs (black line), this behavior does generalize across the song set. The shaded quartile area suggests that this behavior is more consistent for onset of vocals than onset of chorus. In comparison, histogram slopes from randomly selected 15-s windows, shown in the bottom plot, do not reach the percentile levels of the musically salient conditions.

### 3.4. Sample size for consistent query offset distributions

**Figure 6:**
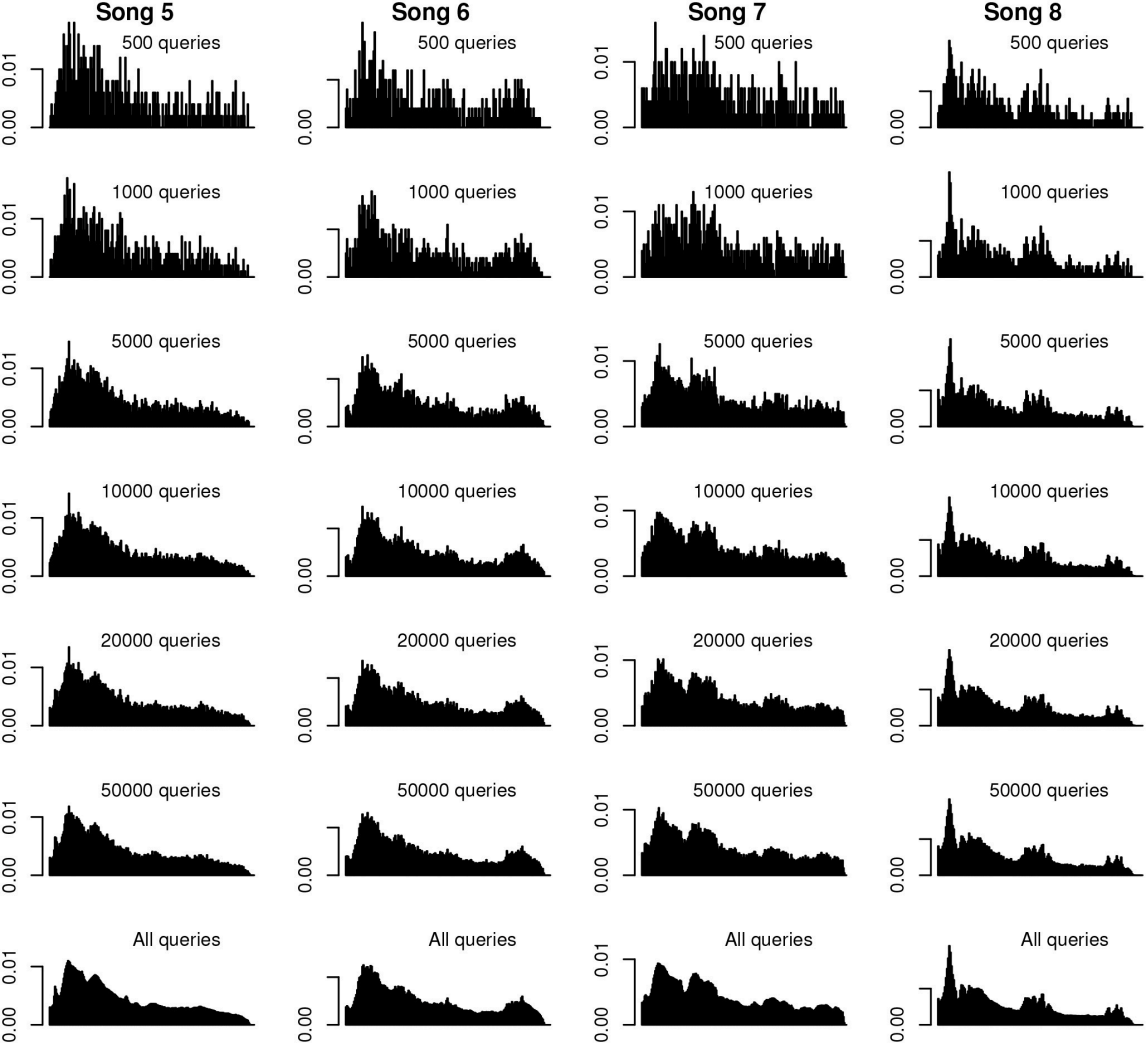
**Example subsampled histograms**. Histograms (density plots) of various quantities of random subsamples for four of the songs. Histograms are scaled to common density and time axes on a per-song basis. The most prominent peaks of the full-sample histogram emerge with as few as 1,000 queries, and are visually similar by 20,000 queries. The finer details of the full-distribution histograms are discernible with subsamples of 50,000 queries.

Our final question concerns the necessary data size to reach a “consistent” distribution of offsets. Figure 6 shows histograms of random subsamples of varying amounts for four of the songs in our set (subsampled histograms for the remaining songs can be found in Figures S5–S8). As can be appreciated by visual inspection of the plots, main peaks in offset distributions are fairly well separated from noise with as few as 1,000 queries. Based on observation, we consider a sample of 20,000 adequate to represent the general shape of the overall distribution, with finely temporally resolved peaks emerging when 50,000 queries are used.

**Figure 7:**
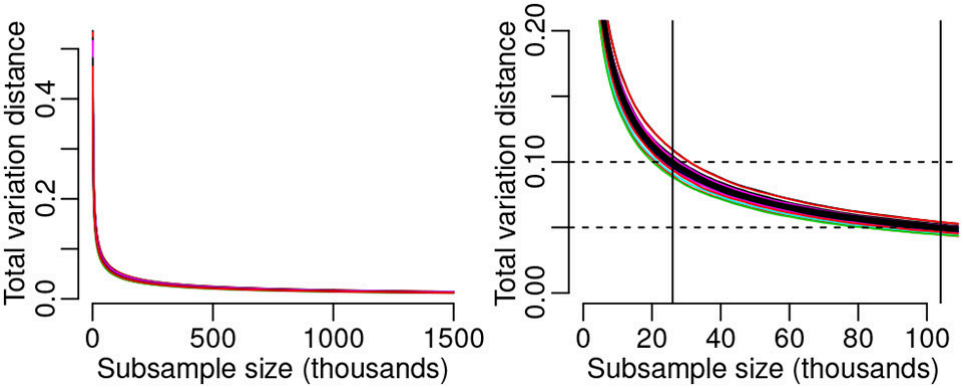
**Histogram consistency as a function of data size**. **(Left)** Median of per-song total variation distance computed across the songs, as a function of subsample size in each of the two distributions being compared. Results of individual songs (colored curves) lie close to the median. Total variation distance shows a sharp drop for subsample sizes up to around 200,000 observations followed by a gradual decrease to a subsample size of 1.5 million. **(Right)** The median subsample size corresponding to a total variation distance of 0.1 is 26,000 observations. Median total variation distance of 0.05 is attained with a subsample size of 104,000 queries.

The median total variation distance between randomly sampled disjoint subsets as a function of subsample size across the song set is shown in Figure 7. As shown in the left panel (Figure 7), the trajectory of these results is consistent across songs. The distance between distributions of two disjoint subsamples for a given song decreases rapidly as a function of sample size, leveling off well below 500,000 queries. While there exists no standard metric of “good” total variation distance, we identify the median subsample size necessary to achieve total variation distance of 0.1 and 0.05 (Figure 7, right panel). A median subsample size of 26,000 queries is required to achieve total variation distance of 0.1—somewhat in line with our observations of the histograms in Figure 6—while 104,000 queries correspond to a median total variation distance of 0.05.

## 4. Discussion

In this study, we investigated music discovery behavior on a large scale by analyzing the timing of Shazam queries during popular songs. Using a dataset of over 188 million queries of 20 hit songs, our findings suggest a relationship between musical events and the timing of Shazam queries. We show that query offsets are not uniformly distributed throughout a song, but rather vary over the course of a song, and may thus be driven in particular by salient musical and structural elements of the song. Furthermore, the shapes of the offset histograms themselves change over the course of the hit song life cycle, showing that the musical content that compels listeners to query a song changes as a function of song popularity or listener exposure to a song. A closer analysis of salient song parts reveals that the onset of vocals and the first occurrence of the chorus in particular drive an increase in queries. Finally, having ample data, we assessed the consistency of the data as a function of data size, and propose that Shazam query offsets for the present song set reach consistent distributions with around 26,000 queries.

Shazam's user data offer several advantages for the study of music discovery. First and foremost is the scale and scope of the data, representing a massive global user base that performs millions of queries each day. Also, while the current study focused on only a small set of songs, Shazam's music catalog contains over 30 million deduplicated tracks. Thus, in terms of both size and demographic diversity of the experimental sample (users), as well as number of stimuli (song catalog), Shazam data capture music discovery at a scale not attainable in controlled studies. The dataset analyzed here is comparable in size to other recently released industrial datasets for music research. For example, the #nowplaying dataset currently exceeds 56 million tweets (Zangerle et al., 2014), while Gracenote's GNMID14 dataset exceeds 100 million music identification matches (Summers et al., 2016). Shazam data are also ubiquitous, meaning that they reflect music discovery in a variety of contexts throughout daily life. As a result, the user data reflect a wide range of music discovery scenarios. Third, Shazam data possess an ecological validity lacking in controlled laboratory studies, as users engage the application in real-world information-seeking scenarios, and were not asked to adopt this behavior as part of a study. Finally, what uniquely differentiates Shazam's data from most other data—including other large-scale social media data—is its objectivity. By this, we mean that under the assumed primary use case of learning the identity of a musical excerpt, Shazam queries are motivated by interest in some aspect of the musical content, even while the queried excerpt may be unknown to the user. Therefore, interest in musical content may be reflected more directly in Shazam queries than in other formats such as tweets, where the content of a posted tweet (and decision whether to post it) has been mediated by the user, reflecting a confluence of musical taste and the user's conscious awareness of how the posted content aligns with his or her expressed identity (Lonsdale and North, 2011; Rentfrow, 2012).

### 4.1. Musical correlates of Shazam queries

#### 4.1.1. Query volume varies throughout a song

In our first analysis, we tested the uniformity of the offset histograms. Visual inspection of the offset histograms of our song set (Figure 2) and results of statistical tests indicate that the query offset distributions are not uniform, and that queries are more likely to occur at some points during the songs than others. In this way, Shazam query offset histograms may facilitate the “locate” research proposed by Honing (2010), in that they reveal points in a song that a number of listeners found engaging.

The timing and heights of histogram peaks vary from song to song. We surmised that this was a reflection of the variation in song structure (e.g., arrangement of choruses, verses, and other elements) across the song set, but that the peaks might reflect structurally salient events that occur across the songs. By analyzing regions of the histograms time-locked to such events, we were able to show that the initial onset of vocals and occurrence of the first chorus drive increases in query volume—represented by high percentiles of histogram slopes—in a consistent fashion across songs.

**Figure 8:**
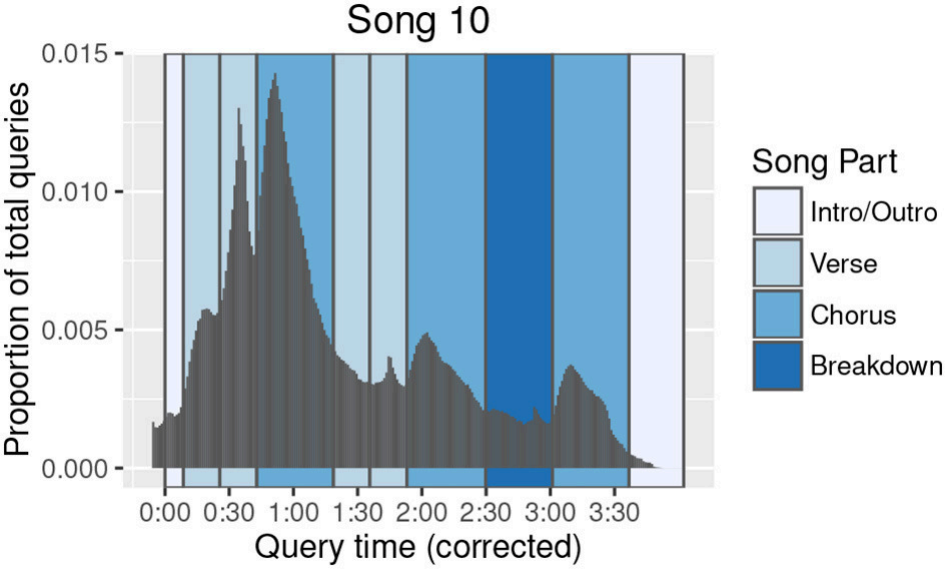
**Song 10 query offset histogram annotated with song parts**. The query offset histogram (density plot) of Song 10 is plotted with structural segmentation annotations. The entire distribution has been shifted back in time by 6 s to demonstrate better alignment of the histogram shape with structural segmentation boundaries once an estimated intent-to-action query latency is considered. Prominent peaks in the histogram are now seen to correspond to specific song parts.

In relating offset histogram peaks to musical events, it is important to keep in mind that users are assumed to successfully query a given broadcast of a song only once. This is reflected to some extent in the overall downward trend in query volume over the duration of a song. Musical content driving Shazam queries may be better characterized, then, as the *first* content in a song that compelled a user to take action and perform the query. Therefore, this content was presumably more engaging than content that came before, but not necessarily more engaging than content that comes after—the user just would not need to query the song a second time, as he had already received the benefit of the query result. Under this reasoning, songs for which the highest histogram peak is not the first peak (for example, Song 14, Song 19, and Song 20) may be of particular interest, as these represent a break from the conventional histogram shape, and may highlight especially engaging musical material occurring later in the song. Furthermore, as shown in Figure 8, histogram peak heights can vary even across occurrences of the same song part (here, most notably for the second verse compared to the first), which may reflect changes in texture, instrumentation, or other musical content. Finally, our present analysis used histogram slopes as indicators of upcoming histogram peaks; future analyses could utilize other histogram features, such as the density or timing of the peaks themselves, or the local minima interspersed between the peaks.

#### 4.1.2. Inferring intent-to-query time

A Shazam query typically does not occur at the exact moment the user was compelled to perform the query. In many cases, the user must retrieve his or her mobile device, unlock it, and load the Shazam application before the query can be performed. Therefore, there exists in the offset data an unknown latency between intent-to-query and query time, which can range from 0 to 10 s or more. We did not attempt to estimate or correct for this latency in our present analyses. However, the histogram slopes following salient musical events may provide some insight into the duration of this delay. If our musical events of interest in fact drive increased queries, we might interpret the time point after such events, at which histogram slopes are consistently high across songs, as an estimate of the mean latency between onset of the song part and initiation of the query. Based on the present results (shown in Figure 5), histogram slopes become consistently high around 9 s after the onset of vocals or the first chorus.

We find that peaks and troughs of an offset histogram are better aligned with structural segmentation boundaries of the song when the histogram is shifted to account for an estimated latency. For example, Figure 8 shows the offset histogram for Song 10, with structural segmentation boundaries visualized in the background. When all offsets are shifted back by 6 s as shown in the figure, the resulting histogram aligns well with the structural segmentation boundaries. Visualizing the other songs in a similar fashion reveals some variation in adjustments required to optimally align histograms with song part boundaries.

Even so, the assumption that histogram slope percentiles or minima convey the intent-to-action delay remains speculative at this stage. Furthermore, the histogram slopes over our time window of interest vary from song to song, as does the optimal time shifting of histograms to align local minima with song-part boundaries. Therefore, additional research—perhaps in a controlled experimental setting—will be required to better characterize this delay, and to determine whether our current proposed approaches for inferring it are appropriate.

#### 4.1.3. Impact of hit song life cycle

As shown in our second analysis, the shapes of offset histograms change over the life cycle of the hit songs in our song set. As a song attained and receded from its peak position on the Billboard chart, queries tended to occur closer to the start of the song. Therefore, even though the underlying musical content was unchanged, users tended to query the audio earlier once a song became successful. As we will later discuss, the intent of the query may have changed, e.g., users querying later in the life cycle may have been doing so for reasons other than to learn the identity of the song. However, it may also be that repeated exposures to such popular songs, which—even while the identity of the song may remain unknown—enhance familiarity, processing fluency, and even preference (Nunes et al., 2015), could compel the user to query the song earlier than he would have done prior to so many exposures. Therefore, it would be interesting to repeat this analysis with songs that never achieved ubiquitous broadcast and widespread popularity, in order to assess in finer detail the impact of popularity and exposure on changes in music discovery behavior.

In interpreting the changes in histogram shape over a song's life cycle, we note that the earliest and latest subsets of data (release date and end date) are always disjoint, but that repeated observations may exist with either of these subsets and the Billboard peak date subset—for example, if a song peaked on Billboard soon after its release.

#### 4.1.4. Disentangling discovery and preference

Under the premise that Shazam queries are primarily searches for identities of unknown songs, it would be erroneous to equate a user's Shazam history with his or her most-loved music. However, if we may assume that users query songs because they are in some way attracted to, or at least aroused by, the songs' musical content, we may conclude that musical attributes of a user's queried songs reflect, to some extent, the musical preferences of that user. In other words, a queried song's musical content, especially around the query offset, may contain features that compel the user to take action and want to know more. In this sense, one's discovered music, more so than freely chosen songs, may be more widely representative of musical preferences, as it encompasses music (and musical features) beyond the scope of what a user could have articulated in advance that he wanted to hear—and possibly across a broader range of musical genres. And, given that known recommended tracks have been shown to be received more positively by listeners than unknown recommendations (Mesnage et al., 2011), music discovery data may be especially valuable in deepening our understanding of positive reception of new music, since it largely reflects music that was both unknown to, and positively received by, the user.

#### 4.1.5. Inferring user intent

While the typical Shazam use case is assumed to be the identification of an unknown audio excerpt, this is by no means the only use case of the service. Other use cases include querying a song in order to access other features of the query result, including the music video, lyrics, artist information; to purchase the song or add it to a third-party playlist; to establish a static access point for the song; to share the song via messaging or social media services; or to demonstrate or test the performance of the application. The shift in query offsets toward the beginning of songs that have peaked in popularity could thus reflect a change in user intent, whereby fewer users are using Shazam to learn the identity of the song at that point, and are instead reflecting an alternative use case.

In fact, in the realm of web searches, informational need is known to account for <50% of queries, with navigational (attempting to reach a specific site) and transactional (reaching a site where further interactions will take place) thought to account for the remainder of use cases (Broder, 2002). This framework of query intent has more recently been extended to the case of multimedia search, for example text queries for videos (Hanjalic et al., 2012). The Shazam use cases mentioned thus far could arguably be categorized as informational (e.g., learn song identity, information about a song) or transactional (e.g., add song to Spotify playlist). However, user intent is not always communicated clearly in a query, and in fact may not even be clear to the user as the query is being performed (Kofler et al., 2016). In the case of Shazam, audio queries are invariant—all initiated by a button press—and therefore provide no insight into user intent. However, it could be possible to infer intent through other factors, such as day or time of query, geography, song popularity, or previous users' interactions with the query result, and to adjust the content of the query result accordingly.

### 4.2. Considerations

While the dataset used in the present study provides several advantages for studying music discovery on a large scale, there exist several unknown contextual factors underlying the queries. First, as our analysis takes into account only query offset and date, we gain no insights from the time or location of the queries. Furthermore, from the present data we do not know how the user reacted to the query result, or whether the query reflects positive reception of the musical content.

In addition, Shazam's utility varies according to the music listening setting. Streaming services and personal playlists provide ubiquitous metadata, which can be accessed with often greater ease than performing a Shazam query. Therefore, Shazam is likely used primarily to identify unknown songs in settings where the user does not otherwise have easy access to song metadata. This could include radio listening as well as public settings in which the user does not control music play (e.g., club, retail, or restaurant). While streaming and playlist listening scenarios typically involve “zero-play” music consumption—that is, the song is likely heard from its start (Frank, 2009)—in radio and other Shazam-worthy settings, we cannot assume the user was exposed to the song from its onset, which could affect the interpretation of some of the present results.

Issues related to the performance of the application should be noted as well. Spurious observations were addressed to some extent during data cleaning, but likely persist throughout the data. Due to a pre-recording functionality of Shazam that begins at application launch, time stamps of query offsets may precede the time of the actual query by up to 3 s for an unknown percentage of users. Certain listening environments, such as those with heavy reverberation, can impede the performance of the application and could therefore require multiple query attempts in order to obtain a result. The presence of vocals during a song may also complicate interpretation of results. While we might interpret a connection between vocals and increased queries as a reflection of musical engagement, it could also be the case that portions of the song with highly prominent vocals may be easier for the Shazam algorithm to match successfully. Prominent vocals may also be easier for a human listener to pick out in a noisy environment. Therefore, disentangling “vocalness” from “catchiness” (by which we mean engaging in the moment, not necessarily memorable in the long term; Burgoyne et al., 2013) could be a useful topic for future research.

In sum, conclusions from the current study must be taken in the context of various unknowns pertaining to users, listening settings, application performance, and other uncontrolled factors. The research questions addressed here could therefore benefit from further investigation in a human-subjects laboratory study setting, where potential confounds and unknowns can be controlled.

### 4.3. Future work

#### 4.3.1. Hooks and catchiness

Through an analysis of offset histogram slopes, this study provides first insights into Shazam queries following song starts, initial onsets of vocals, and first occurrences of choruses. This approach could be broadened to consider more generally the role of “hooks” in music discovery. Musical hooks are defined in many ways, largely describing the part(s) of a song that grab the listener's attention and stand out from other content (Burns, 1987). Hooks need not be restricted only to popular music (Mercer-Taylor, 1999), but are often discussed in the context of popular songs and are thought to occur primarily at structural segmentation boundaries (i.e., starts of song parts; Burns, 1987; Mercer-Taylor, 1999; Burgoyne et al., 2013). The construction of a hook can involve musical features such as rhythm, melody, and harmony, as well as production decisions such as editing and mix (Burns, 1987). The study of musical hooks historically involved human analysis of hand-picked excerpts (Mercer-Taylor, 1999; Kronengold, 2005); in recent years, computational approaches have also evolved (Burgoyne et al., 2013; Van Balen et al., 2013, 2015), which may facilitate hook research over large audio corpuses.

Singability is considered to be a characteristic of hooks (Kronengold, 2005), and is thought to increase listener engagement, both by increasing familiarity and by inspiring the listener to sing along (Frank, 2009). In addition to such intrinsic factors as singability or catchiness, the arrangement of structural elements within a song is also critical to engaging the listener (Mercer-Taylor, 1999). Shazam query offset histograms could prove useful in exploring all of these topics further. While we used annotated lyrics to guide our identification of salient song parts, future research could consider computational models of catchiness—perhaps constructed from computationally extracted audio features (McFee et al., 2015),^7^ higher-level musical features (Van Balen et al., 2015),^8^ and structural segmentation boundaries (Nieto and Bello, 2016)^9^—and use Shazam query distributions to validate the models. Alternatively, a model could be learned directly from features of the audio corresponding to the histogram peaks themselves. In addition to increasing our understanding of what types of musical features attract listeners, these analyses have the potential to explain the appearance of higher histogram peaks later in a song, as in Song 10 (Figure 8).

#### 4.3.2. Modeling and prediction of hit songs

Large-scale music discovery data may also provide new insights into modeling and predicting hit songs. Hit prediction remains an open area of research (Pachet and Roy, 2008; Pachet, 2012), and has been attempted with audio and lyrics features (Dhanaraj and Logan, 2005; Herremans et al., 2014) and Twitter data (Kim et al., 2014; Zangerle et al., 2016) with varying success. Other recent studies have found instrumentation (Nunes and Ordanini, 2014) and lexical repetition (Nunes et al., 2015) to be predictive of peak chart position for past Billboard hits. The potential of Shazam's data for hit prediction has been discussed in news articles.^10^ Audio, lyrics, instrumentation, and other features found to be predictive of success in the past studies mentioned above could be explored using query offset histograms. While the present analysis considered only hit songs, query offsets—or other Shazam data attributes—of a song set with more variation in popularity could lead to the formulation of unique predictors of eventual song success.

#### 4.3.3. Other time-based analyses

When thinking about Shazam queries, time can signify many things. Our present analyses considered two types of time: The timing of queries over the course of a song, and the longer-term time scale of the hit song life cycle, spanning several months. Other approaches to time could include day of week—known to impact listening behavior (Schedl, 2013) as well as Shazam query volume—and time of day.

#### 4.3.4. Other behaviors and data attributes

The present study provides novel insights into music discovery, using only two of Shazam's many data attributes. A variety of additional musical questions could be addressed using Shazam user data. User interactions with the application after receiving a query result could provide insight into user preference and user intent. Other analyses could model music discovery or preference by considering specific geographies, musical genres, or even individual users. Large-scale data have been used to address specific musical questions including the long tail in music-related microblogs (Schedl et al., 2014), social media behavior of Classical music fans (Schedl and Tkalčič, 2014), the relationship between musical taste and personality factors (Bansal and Woolhouse, 2015), and Twitter activity around a specific musical event (Iren et al., 2016). Using Shazam data in this way—to address specific musical questions—promises interesting approaches for future research endeavors.

## Author contributions

Conceived and designed the research: BK, FR, CB, JB. Aggregated the data: BK, CB. Analyzed the data: BK, FR. Wrote the paper: BK, FR, CB, JB.

## Funding

This research was supported by the Wallenberg Network Initiative: Culture, Brain, Learning (BK, JB), the Roberta Bowman Denning Fund for Humanities and Technology (BK, JB), Shazam Entertainment, Ltd. (BK, CB), and the E. K. Potter Stanford Graduate Fellowship (FR).

### Conflict of interest statement

Authors BK and CB are present or former paid employees of Shazam Entertainment, Ltd. Authors FR and JB declare that the research was conducted in the absence of any commercial or financial relationships that could be construed as a potential conflict of interest.

## Footnotes

^1^http://www.shazam.com.

^2^Shazam also has a desktop application for Mac.

^3^http://www.billboard.com/charts/year-end/2015/hot-100-songs.

^4^http://genius.com.

^5^http://genius.com/static/licensing.

^6^http://purl.stanford.edu/fj396zz8014.

^7^https://github.com/librosa/librosa.

^8^https://github.com/jvbalen/catchy.

^9^https://github.com/urinieto/msaf.

^10^http://www.theatlantic.com/magazine/archive/2014/12/the-shazam-effect/382237/.
