# Supplementary material for: Characterizing Listener Engagement with Popular Songs Using Large-Scale Music Discovery Data
Source: Front Psychol. 2017 Mar 23;8:416. doi: 10.3389/fpsyg.2017.00416 (PMC5362644; doi:10.3389/fpsyg.2017.00416)
Supplement: Supplementary file 1 [file DataSheet1.PDF]

Supplementary Material:
Characterizing Listener Engagement with Popular Songs Using Large-Scale Music Discovery Data

Blair Kaneshiro*, Feng Ruan, Casey W. Baker and Jonathan Berger
*Correspondence:
Blair Kaneshiro:
blairbo@ccrma.stanford.edu

1 SUPPLEMENTARY TABLES AND FIGURES

1.1 Figures

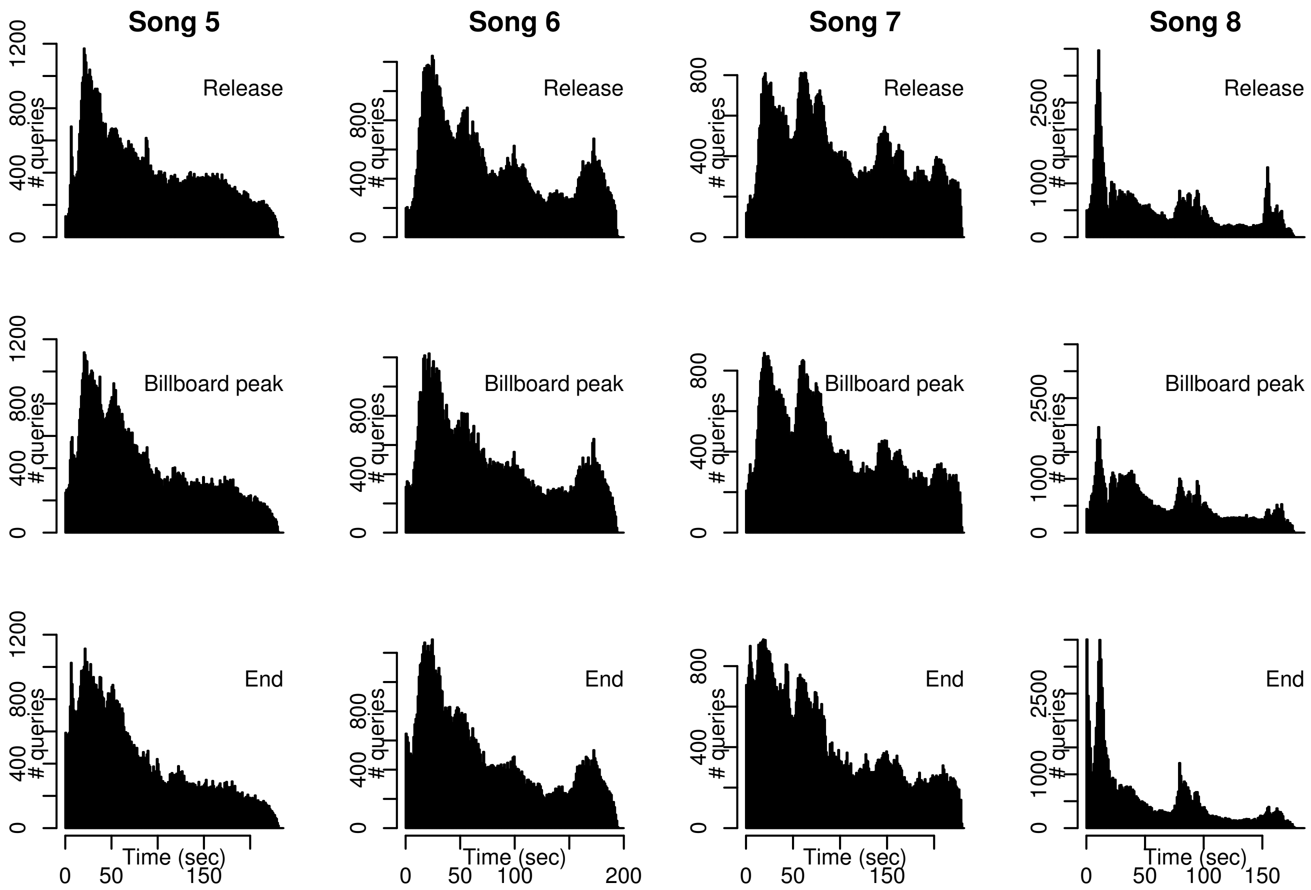

Figure S1. Histograms throughout hit song life cycle, Song 5 through Song 8. Subsampled query distributions at the time of song release, date of peak position on Billboard, and end of the dataset.

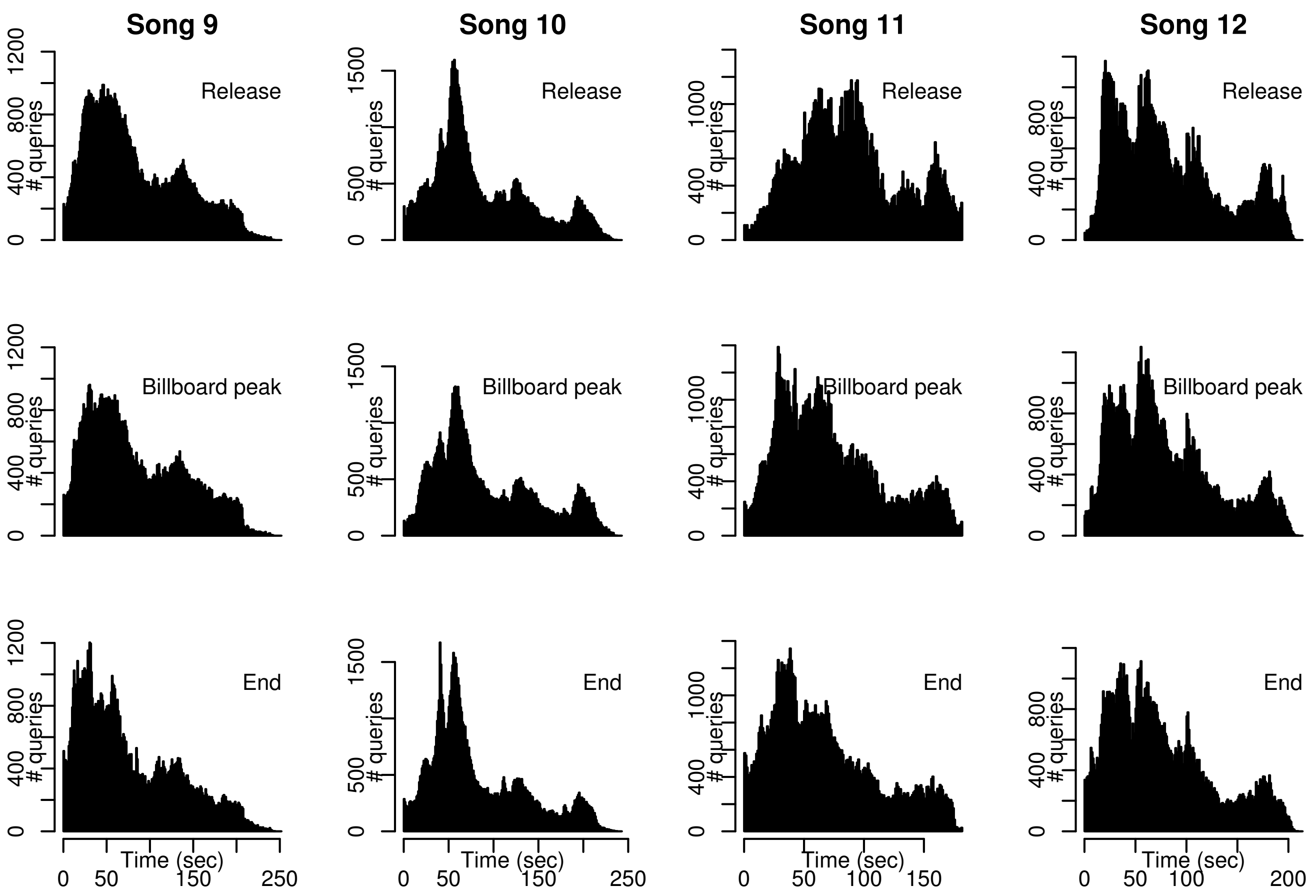

Figure S2. Histograms throughout hit song life cycle, Song 9 through Song 12. Subsampled query distributions at the time of song release, date of peak position on Billboard, and end of the dataset.

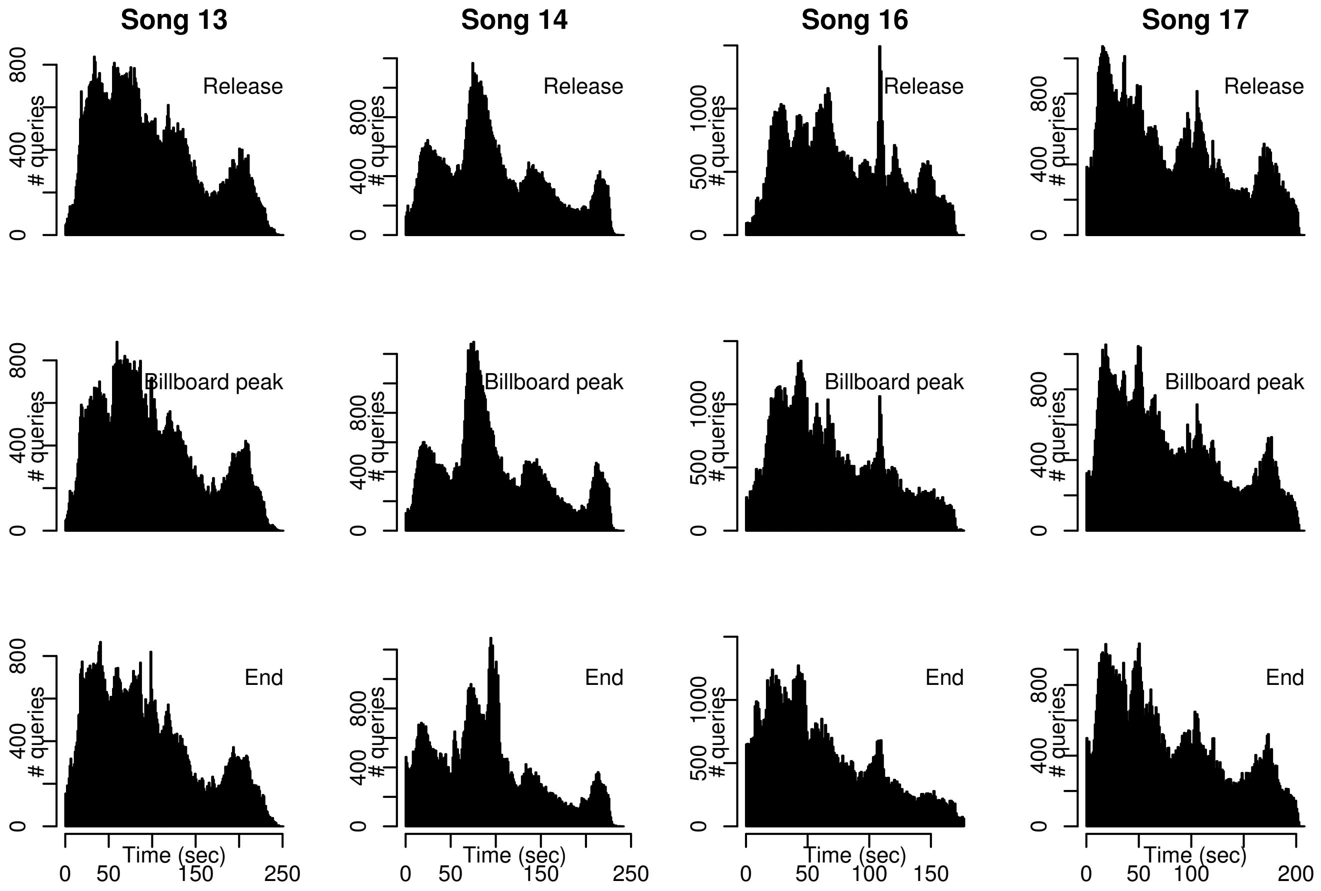

Figure S3. Histograms throughout hit song life cycle, Song 13 through Song 17. Subsampled query distributions at the time of song release, date of peak position on Billboard, and end of the dataset. Song 15 is omitted from analysis.

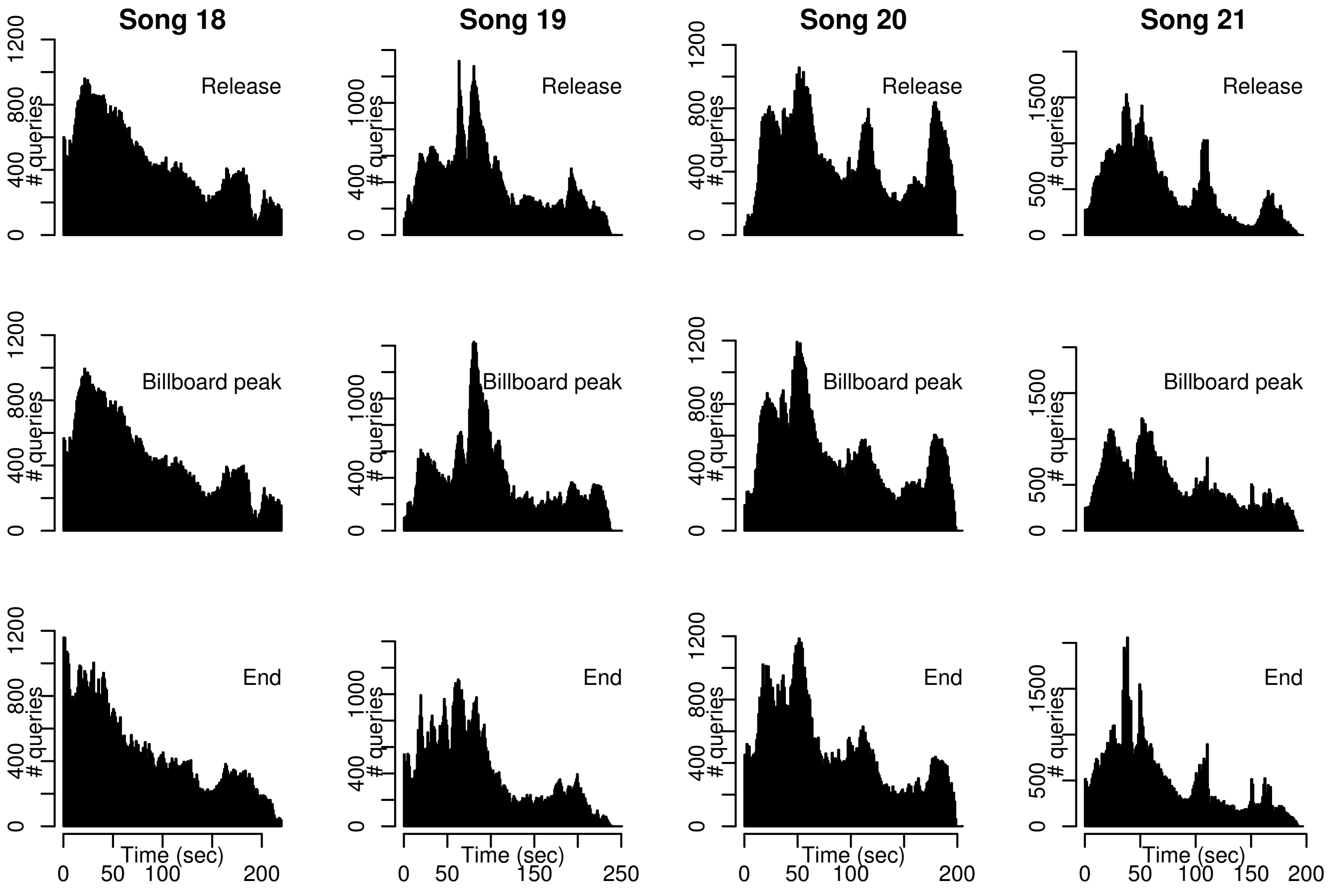

Figure S4. Histograms throughout hit song life cycle, Song 18 through Song 21. Subsampled query distributions at the time of song release, date of peak position on Billboard, and end of the dataset.

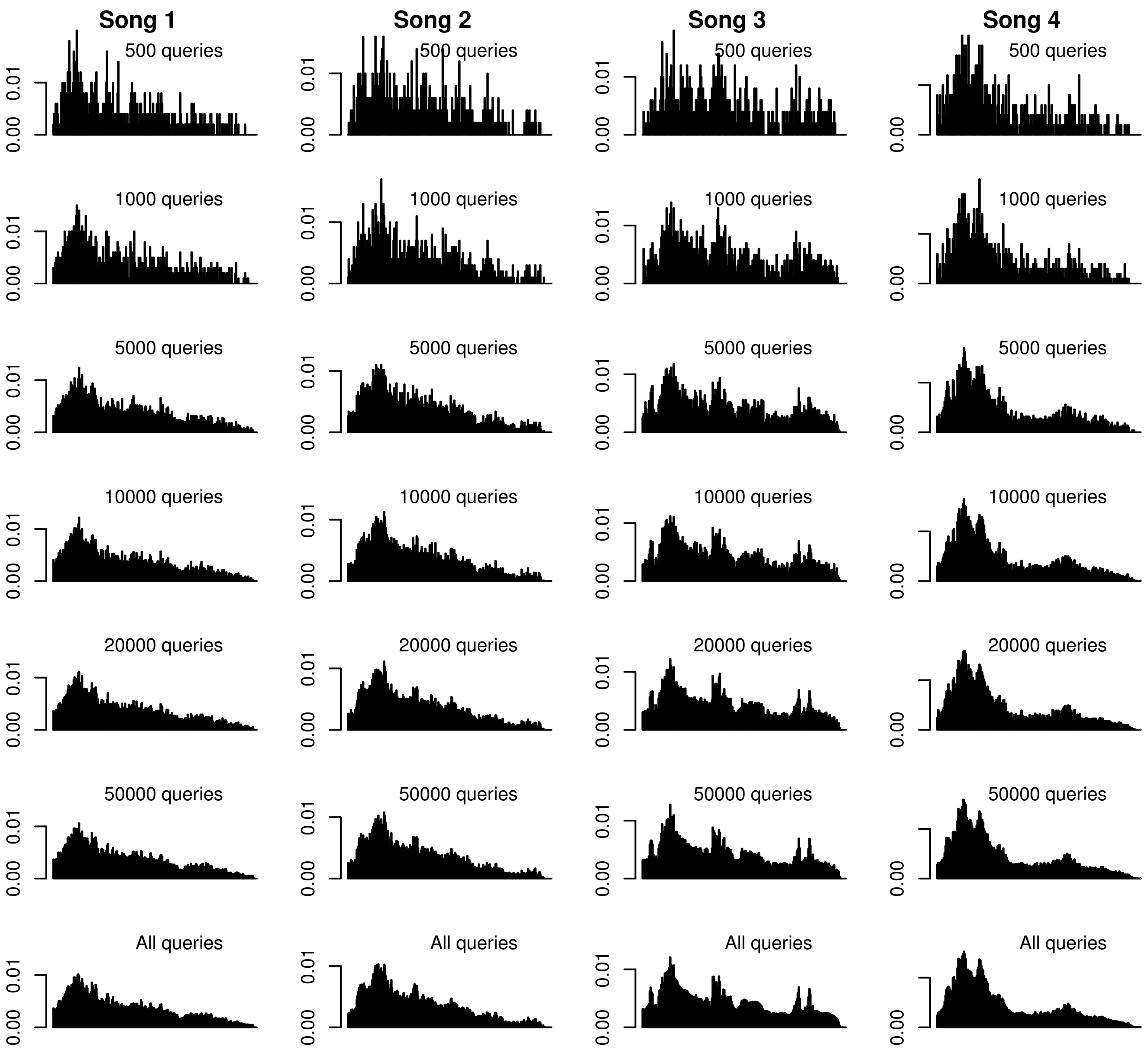

Figure S5. Example subsampled histograms, Song 1 through Song 4. Histograms (density plots) of various quantities of random subsamples. Histograms are scaled to common density and time axes on a per-song basis.

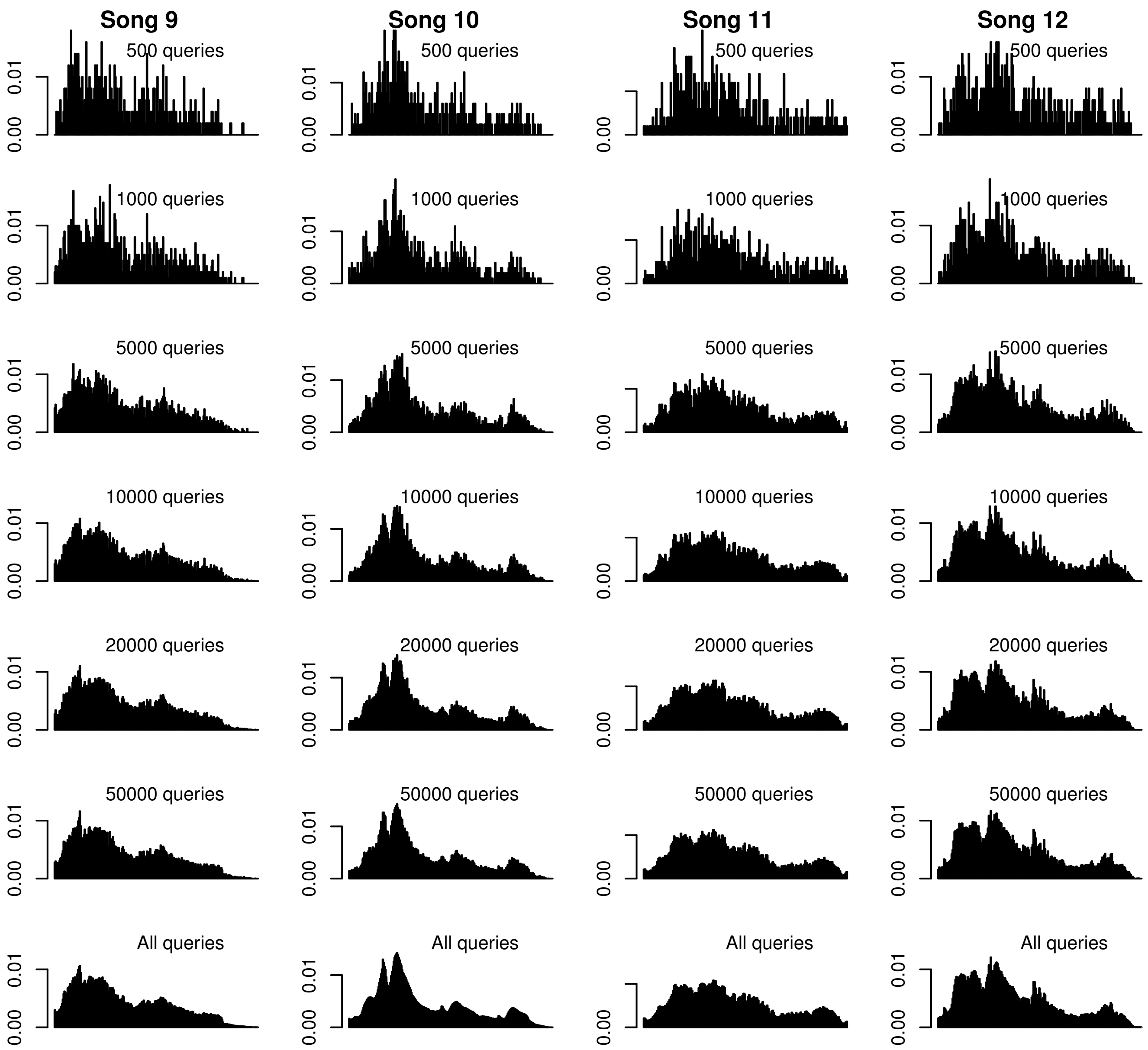

Figure S6. Example subsampled histograms, Song 9 through Song 12. Histograms (density plots) of various quantities of random subsamples. Histograms are scaled to common density and time axes on a per-song basis.

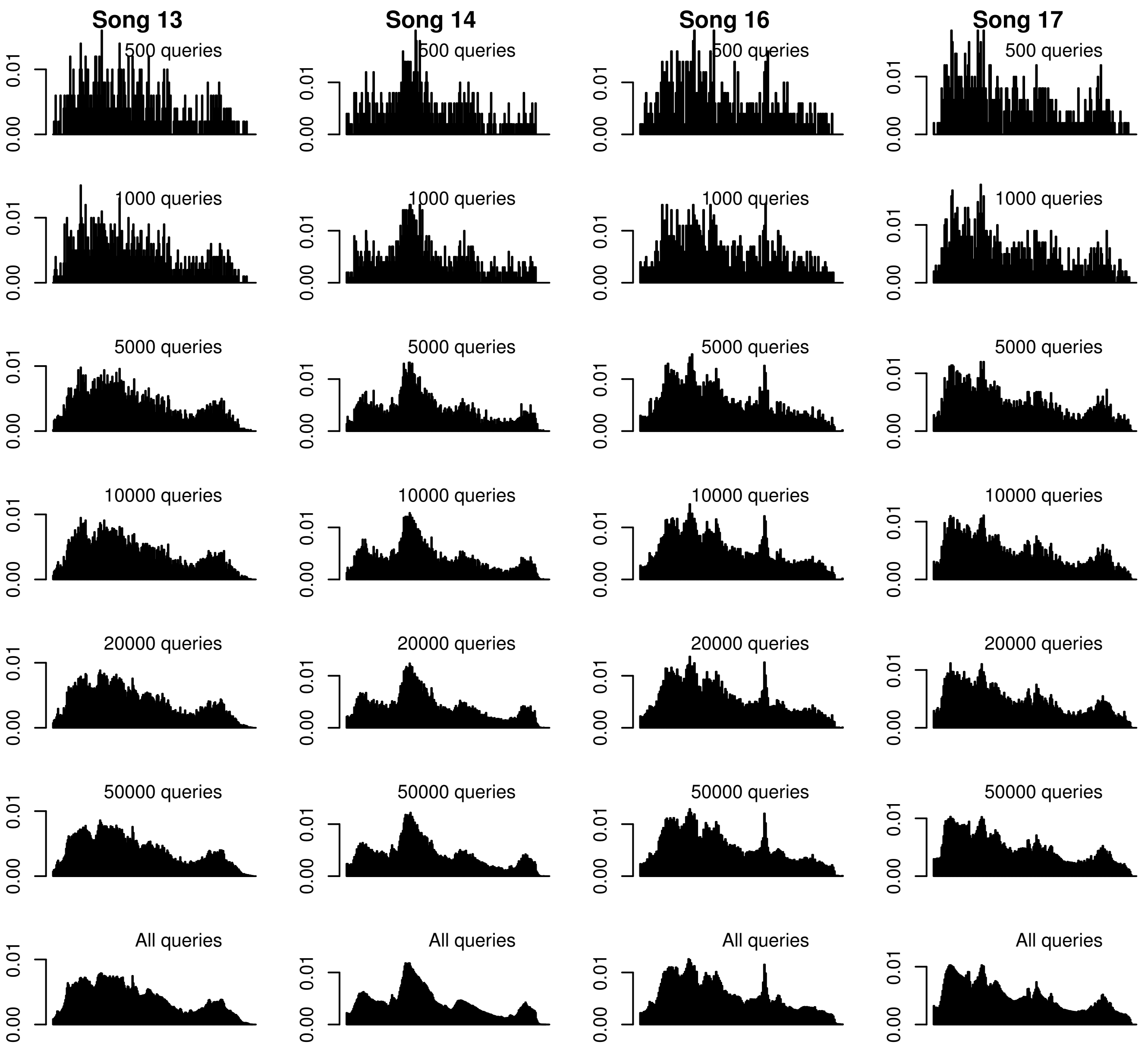

Figure S7. Example subsampled histograms, Song 13 through Song 17. Histograms (density plots) of various quantities of random subsamples. Histograms are scaled to common density and time axes on a per-song basis. Song 15 is omitted from analysis.

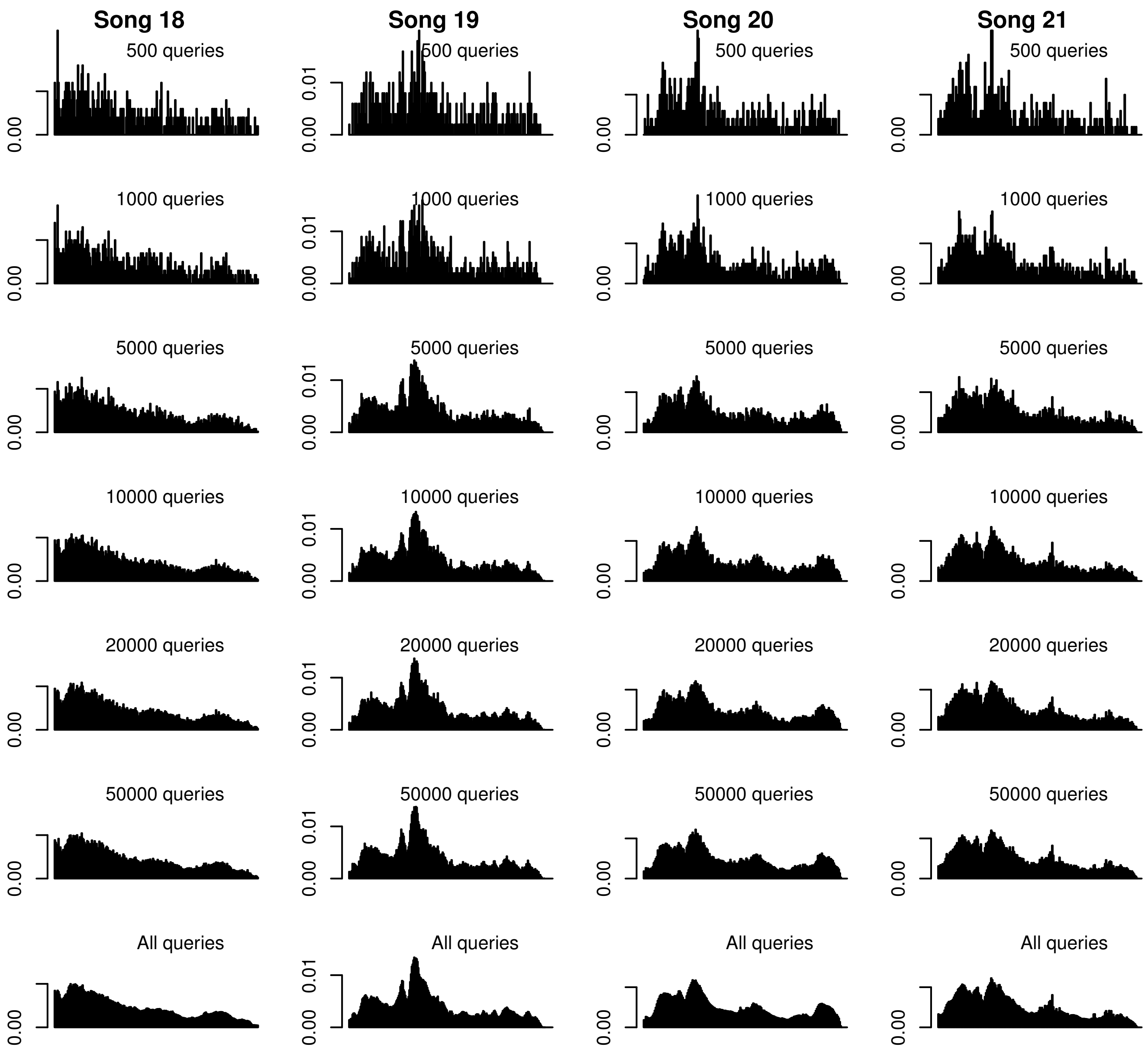

Figure S8. Example subsampled histograms, Song 18 through Song 21. Histograms (density plots) of various quantities of random subsamples. Histograms are scaled to common density and time axes on a per-song basis.

1.2 Tables

	Track identifiers		Release and peak Billboard dates				Song parts	
**Rank**	**Shazam**	**Amazon ASIN**	**Release**	**Peak BB**	**Rel-to-BB**	**BB-to-end**	Vocals	Chorus
**1**	160157606	B00S226A48	2014-11-10	2015-01-17	68	500	17	50
**2**	123453088	B00JLJ6VSQ	2014-09-24	2015-01-31	129	486	1	71
**3**	235243031	B00TJ69DHM	2015-03-17	2015-04-25	39	402	10	10
**4**	119440654	B00QSDA4IG	2014-04-22	2015-05-16	389	381	15	55
**5**	144086612	B00XDI1GEU	2015-01-13	2015-03-28	74	430	8	39
**6**	150773872	B00P6Y1OJG	2014-09-10	2015-05-30	262	367	3	3
**7**	157666207	B00OXE38D0	2014-11-10	2014-11-29	19	549	5	45
**8**	229572648	B00X5MVZ6I	2015-05-05	2015-07-18	74	318	13	13
**9**	221708306	B00S17FWPW	2014-12-23	2015-05-02	130	395	13	59
**10**	267041500	B014DIAKRM	2015-05-27	2015-10-03	129	241	8	42
**11**	119205187	B01HPWYZTC	2014-05-19	2015-07-25	432	311	15	40
**12**	269065410	B014DIAVS0	2015-06-08	2015-08-22	75	283	8	44
**13**	221827125	B00S17FZ7C	2015-01-07	2015-03-07	59	451	20	60
**14**	92719600	B00M01JCAQ	2013-09-13	2014-12-20	463	528	0	56
**16**	234782921	B00YIAXFG4	2015-03-02	2015-08-29	180	276	10	30
**17**	238644669	B00VM6SI5K	2015-03-09	2015-06-20	103	346	1	36
**18**	147771254	B00OXE3GEG	2014-08-18	2014-09-06	19	633	5	41
**19**	236350641	B00U0AIECQ	2015-02-27	2015-07-18	141	318	20	68
**20**	154437676	B0189VVN5W	2015-02-19	2015-08-29	191	276	4	37
**21**	228968059	B010DROMFI	2015-06-29	2015-10-31	124	213	10	39

Table S1. Additional song metadata. Rank: The Billboard ranks of the songs in the song set. Track identifiers: Shazam track identifiers can be appended to the URL `http://www.shazam.com/track/` to reach the track's Shazam webpage. The ASIN (Amazon Standard Identification Number) can be used as a search term in Amazon to reach the page of the digital track purchased and analyzed in the study. Release and peak Billboard dates: Release dates were retrieved from each song's Wikipedia page. Peak Billboard dates were retrieved from the Billboard Hot 100 weekly online charts. Rel-to-BB lists the number of days between the release date and peak Billboard date. BB-to-end lists the number of days between the peak Billboard date and final date in the dataset (May 31, 2016). Song parts: Vocals and chorus elements were identified using song-part-annotated lyrics from the online service Genius. Values are floored times, in seconds, of the start of the song part in the audio.
